# Patterns of behaviors related to modifiable health risk factors among Brazilian older adults: Data from the 2013 and 2019 National Health Surveys

**DOI:** 10.1590/1980-549720260042

**Published:** 2026-08-17

**Authors:** Mariana Sousa de Abreu Menezes, Zayanna Christine Lopes Lindôso, Ana Karina Teixeira da Cunha França, Poliana Cristina de Almeida Fonseca Viola, Bruno Luciano Carneiro Alves de Oliveira

**Affiliations:** I Universidade Federal do Maranhão, Graduate Program in Public Health – São Luís (MA), Brazil.; II Universidade Federal de Pelotas, Occupational Therapy Undergraduate Program Council – Pelotas (RS), Brazil.

**Keywords:** Aged, Healthy aging, Social determinants of health, Latent class analysis, Combined risk behaviors

## Abstract

**Objective:**

To analyze patterns of modifiable health risk behaviors among Brazilian older adults (≥60 years) using data from the 2013 and 2019 National Health Surveys.

**Methods:**

A cross-sectional study was conducted based on secondary data from the 2013 and 2019 National Health Surveys. Behavior patterns were defined using Latent Class Analysis according to four main domains: smoking, alcohol consumption, physical activity, and diet. Relative frequencies and their 95% confidence intervals (95%CI) were calculated for lifestyle variables, as well as for independent variables associated with socioeconomic and demographic factors.

**Results:**

Five behavior patterns were identified for each year. In 2013, patterns related to unhealthy habits (smoking), physical inactivity, and dietary patterns (including bean, vegetable, legume, and fish consumption) were identified. In 2019, the findings also involved the same patterns found in 2013, with the difference that fish consumption showed greater relevance in differentiating between classes, although its population prevalence decreased over the period. Physical inactivity was associated with harmful habits, including inadequate diet, smoking, and alcohol consumption. Age, sex, education level, and race significantly influenced these patterns.

**Conclusion:**

In Brazil, unhealthy aging overlaps with multiple modifiable risk factors. Understanding these patterns and their determinants enables the development of strategies to reduce health inequalities and promote quality of life and well-being among older adults. A combined approach to risk factors contributes to more effective public policies.

## INTRODUCTION

In 2022, Brazil had approximately 32 million individuals aged 60 years old or older, representing 15.8% of the total population^1^. Projections indicate that by 2030, the number of older adults in the country will exceed that of children and adolescents^2^. However, in 2023, a substantial proportion of this population reported engaging in health-risk behaviors: 56% had a sedentary lifestyle; 14% reported excessive alcohol consumption; 12% smoked; and only 18% reported adequate consumption (fruits and vegetables)^1–4^.

Such behaviors are associated with noncommunicable diseases (NCDs), the leading causes of morbidity and mortality worldwide, which disproportionately affect older adults. The prevalence of NCDs is influenced by a range of modifiable health-risk factors^5,6^. However, health-risk behaviors are often examined in isolation, overlooking their interdependence, co-occurrence, and overlap in shaping distinct health patterns^7,8^. Clusters of health-risk behaviors amplify their detrimental effects by acting synergistically to compromise quality of life, deteriorate overall health status, reduce the likelihood of longevity, and increase the risk of premature mortality^9,10^.

Older adults often exhibit complex patterns of health-risk behaviors shaped by the social determinants of health^11^. These combined behavioral patterns reflect healthy and unhealthy habits adopted throughout the life course, which typically co-occur and are more difficult to modify individually.

Therefore, monitoring health-risk behaviors and their socioeconomic, geographic, and racial determinants can contribute to a better understanding of the effects of lifestyle, NCD management, and the quality of multidisciplinary interventions among older adults. Consequently, nationwide health surveys, such as the 2013 and 2019 National Health Surveys (*Pesquisa Nacional de Saúde* – PNS), provide an opportunity to conduct these analyses within the Brazilian context^12^.

Given this context, this study aimed to analyze patterns of modifiable health-risk behaviors among older Brazilian adults in 2013 and 2019.

## METHODS

### Study type and location

This cross-sectional study was based on secondary data from the 2013 and 2019 PNS. PNS is a population-based, nationally representative household survey conducted jointly by the Brazilian Institute of Geography and Statistics (*Instituto Brasileiro de Geografia e Estatística* – IBGE) and the Brazilian Ministry of Health (MoH).

### Context

PNS aims to present results that are representative of the Brazilian population. To this end, it employs a complex sampling design^13^, organized by clusters, involving three stages of selection: census tracts (primary units), households (secondary units), and adult residents aged 18 years old or older (tertiary units). However, in 2019, at the third stage, the selected resident was aged 15 years old or older^14,15^.

The 2013 PNS employed a sampling design involving interviews conducted in 6,062 primary sampling units (PSUs) and 64,348 households^12^, whereas the 2019 PNS covered 8,031 PSUs and 94,114 households. For the present study, responses from the individual questionnaire completed by older adults (≥60 years of age) were analyzed. The final analytical sample comprised 11,177 older adults from the 2013 PNS and 22,728 from the 2019 PNS.

### Variables

Lifestyle-related variables were organized into four domains: smoking, alcohol consumption, physical activity, and diet, which were identified in Module P of the selected resident's questionnaire.

Dietary intake was assessed based on the weekly consumption of beans, fruits, vegetables, and fish, measured by frequency of consumption per week. These variables were dichotomized according to national and international guideline recommendations; adequate consumption was defined as ≥5 times per week for beans, fruits, and vegetables, and ≥2 times per week for fish. Alcohol consumption was defined based on the number of drinks consumed on a single occasion within the previous 30 days and categorized as excessive consumption when ≥5 drinks for men or ≥4 drinks for women.

Regarding leisure-time physical activity, individuals were classified as physically active if they achieved at least 150 minutes of moderate-intensity activity or 75 minutes of vigorous-intensity activity per week, in accordance with the recommendations of the World Health Organization (WHO). Smoking status was defined by classifying individuals who reported current daily or occasional use of tobacco products as smokers.

The independent variables included sex, age (years), color or race, living with a partner, religion, education, income, and area of residence.

### Statistical analysis

In the descriptive analysis, relative frequencies and their 95% confidence intervals (95% CIs) were calculated for health behaviors and socioeconomic and demographic variables.

To define behavioral patterns, Latent Class Analysis (LCA) was performed, a statistical method that identifies distinct groups based on response patterns observed in categorical variables^16^. LCA was used to identify behavioral patterns related to modifiable risk factors based on categorical lifestyle variables, with estimates calculated separately for 2013 and 2019. During the construction of the latent variable, models with varying numbers of latent classes were developed and evaluated to determine the most appropriate model. Model selection was guided by the Akaike Information Criterion (AIC), Bayesian Information Criterion (BIC), Adjusted Bayesian Information Criterion (aBIC), and log-likelihood, favoring models with lower values for these criteria compared with previous models^16^; the highest entropy value was also considered. The final decision regarding the number of classes considered the interpretability and coherence of the identified patterns.

To test the association between latent classes and independent socioeconomic and demographic variables, adjusted multinomial logistic regression models were estimated. The effect measure used was the odds ratio (OR), with corresponding 95% confidence intervals (95%CIs) and a significance level of p < 0.05. The complex sample design, including strata and PSUs, was accounted for in the statistical analyses. Analyses were performed using RStudio software (version 2023.9.0.463), and the "poLCA" package was used for latent class analysis.

The 2013 and 2019 PNS editions were approved by the National Research Ethics Commission of the Ministry of Health. For the 2013 edition, the approval number was 328,159, and for the 2019 edition, it was 3,529,376.

#### Data availability statement:

The entire dataset supporting the results of this study is available from the corresponding author upon request. The dataset is not publicly available due to the integration of databases performed by the authors.

## RESULTS

The study included 11,177 individuals aged 60 years old or older from the 2013 PNS and 22,728 from the 2019 PNS.

Table 1 presents the socioeconomic and demographic characteristics of the study population in 2013 and 2019. No significant changes were observed in the sex distribution. Women constituted the majority in both 2013 (56.94%; 95%CI 54.8–58.0) and 2019 (56.7%; 95%CI 55.6–57.8). The proportion of White individuals decreased between the two survey years (2013: 53.8%; 95%CI 51.9–55.6; 2019: 50.5%; 95%CI 49.3–51.8; p=0.02). A significant change was observed in educational attainment: the proportion of individuals with incomplete elementary education decreased from 70.7% (95%CI 68.9–72.4) to 63.3% (95%CI 62.0–64.5), whereas the proportion with a completed higher education degree increased from 9.4% (95%CI 8.1–10.7) to 11.3% (95%CI 10.5–12.1; p<0.001). Income distribution also changed, with an increase in the proportion of individuals in the first income quintile (2013: 20.6%; 95%CI 19.2–22.0; 2019: 24.4%; 95%CI 23.4–25.4; p<0.001). Urban areas predominated in both survey years, with no significant changes in the distribution of the area of residence.

**Table 1:** Socioeconomic and demographic characteristics of older adults interviewed in the National Health Survey, 2013 and 2019, Brazil.

Characteristics		95%CI		
		2013 (n=11,177)	2019 (n=22,728)	p-value
Sex				
	Male	43.6 (42.0–45.2)	43.3 (42.2–44.4)	0.76
	Female	56.4 (54.8–58.0)	>56.7 (55.6–57.8)	
Age range				
	60 to 64	32.0 (30.5–33.5)	31.1 (30.0–32.1)	0.72
	65 to 69	24.6 (23.3–25.9)	>25.2 (24.3–26.1)	
	70 to 74	18.3 (17.0–19.5)	>18.1 (17.3–18.9)	
	75 or older	25.2 (23.8–26.5)	>25.6 (24.6–26.5)	
Race/skin color				
	White	53.8 (51.9–55.6)	50.5 (49.3–51.8)	0.02
	Black	9.2 (8.2–10.2)	>10.3 (9.6–11.0)	
	Brown	35.6 (33.9–37.2)	>37.4 (36.2–38.5)	
	Others	1.4 (1.1–1.8)	>1.8 (1.5–2.1)	
Marital status				
	With spouse/partner	53.5 (51.9–55.1)	50.7 (49.5–51.8)	0.004
	Without spouse/partner	46.5 (44.9–48.1)	>49.3 (48.2–50.5)	
Education				
	Up to incomplete primary education or equivalent	70.7 (68.9–72.4)	63.3 (62.0–64.5)	0.001
	Incomplete secondary education or equivalent	8.1 (7.2–9.0)	>9.5 (8.9–10.2)	
	Incomplete higher education or equivalent	11.8 (10.8–12.9)	>15.9 (15.0–16.8)	
	Complete higher education	9.4 (8.1–10.7)	>11.3 (10.5–12.1)	
Income				
	1^st^ Quintile	20.6 (19.2–22.0)	24.4 (23.4–25.4)	0.001
	2^nd^ Quintile	22.7 (21.3–24.1)	>17.4 (16.6–18.2)	
	3^rd^ Quintile	16.6 (15.3–18.0)	>16.1 (15.2–16.9)	
	4^th^ Quintile	21.3 (19.9–22.7)	>21.8 (20.8–22.7)	
	5^th^ Quintile	18.7 (17.1–20.2)	>20.4 (19.3–21.5)	
Religion				
	Yes	50.1 (48.3–51.8)	48.5 (47.4–49.7)	0.15
	No	49.9 (48.2–51.7)	>51.5 (50.3–52.6)	
Area of residence				
	Urban	85.2 (83.7–86.6)	85.5 (84.6–86.3)	0.76
	Rural	14.8 (13.4–16.3)	>14.5 (13.7–15.4)	

Source: Brazil, PNS 2013 and 2019.

The prevalence of modifiable health-risk factors was estimated for both survey years (data not shown). No statistically significant changes were observed in the prevalence of smoking (2013: 12.2%; 95%CI 11.1–13.2; 2019: 11.1%; 95%CI 10.5–11.8) or bean consumption (2013: 71.4%; 95%CI 69.9–72.9; 2019: 70.2%; 95%CI 69.1–71.2; p>0.05). Excessive alcohol consumption increased from 4.2% (95%CI 3.6–4.9) to 5.8% (95%CI 5.2–6.3; p<0.001). The prevalence of leisure-time physical activity also increased significantly, from 13.4% (95%CI 12.3–14.5) to 19.4% (95%CI 18.5–20.4; p<0.001). Likewise, the prevalence of vegetable consumption increased from 38.1% (95%CI 36.3–39.9) to 63.4% (95%CI 62.3–64.5; p<0.001), and that of fruit consumption increased from 55.0% (95%CI 53.3–56.7) to 59.2% (95%CI 58.1–60.3; p<0.001). Conversely, fish consumption decreased from 58.2% (95%CI 56.3–60.1) to 50.0% (95%CI 48.8–51.2; p<0.001).

Table 2 presents the fit statistics used to determine the optimal number of latent classes. The selection of the number of classes was based on statistical criteria (AIC, BIC, aBIC, log-likelihood, and entropy) as well as the substantive interpretability of the classes. AIC, BIC, and aBIC values decreased through the five-class model and remained relatively stable in subsequent models. The final model was selected based on the balance among statistical fit, parsimony, and entropy, while prioritizing solutions that were both interpretable and theoretically plausible.

**Table 2:** Criteria for selecting the number of latent classes formed by older adults in the National Health Survey for the years 2013 and 2019.

Classes	2013							2019						
	Log-likelihood	Residual df	AIC	BIC	aBIC	cAIC	Entropy	Log-likelihood	Residual df	AIC	BIC	aBIC	cAIC	Entropy
2	-39340.91	112	78535.31	78821.65	78773.98	78836.65	0.405	-83557.24	112	167144.5	167264.9	167217.3	167279.9	0.478
3	-39242.70	104	78535.31	78699.80	78626.71	78722.80	0.494	-83296.85	104	166639.7	166824.4	166751.3	166847.4	0.432
4	-39165.64	96	78393.29	78620.26	78521.75	78651.26	0.501	-83190.60	96	166443.2	166692.2	166593.7	166723.2	0.403
**5**	**-39140.54**	**88**	**78357.52**	**78644.63**	**78520.69**	**78683.63**	**0.625**	**-83130.20**	**88**	**166338.4**	**166651.6**	**166527.7**	**166690.6**	**0.47**
6	-39123.83	80	78341.67	78685.79	78536.43	78732.79	0.491	-83109.40	80	166312.8	166690.3	166540.9	166800.0	0.417
7	-39116.85	72	78342.05	78746.39	78571.61	78801.39	0.483	-83096.63	72	166303.3	166745.0	166570.2	166800.0	0.395
8	-39111.47	64	78345.77	78810.21	78610.00	78873.21	0.508	-83096.63	72	166298.7	166745.0	166604.4	166867.6	0.422

AIC: Akaike Information Criterion; BIC: Bayesian Information Criterion; aBIC: Adjusted Bayesian Information Criterion; cAIC: Consistent Akaike Information Criterion.

The selected number of classes is shown in bold.

Source: Brazil, PNS 2013 an 2019.

**Figure 1:**
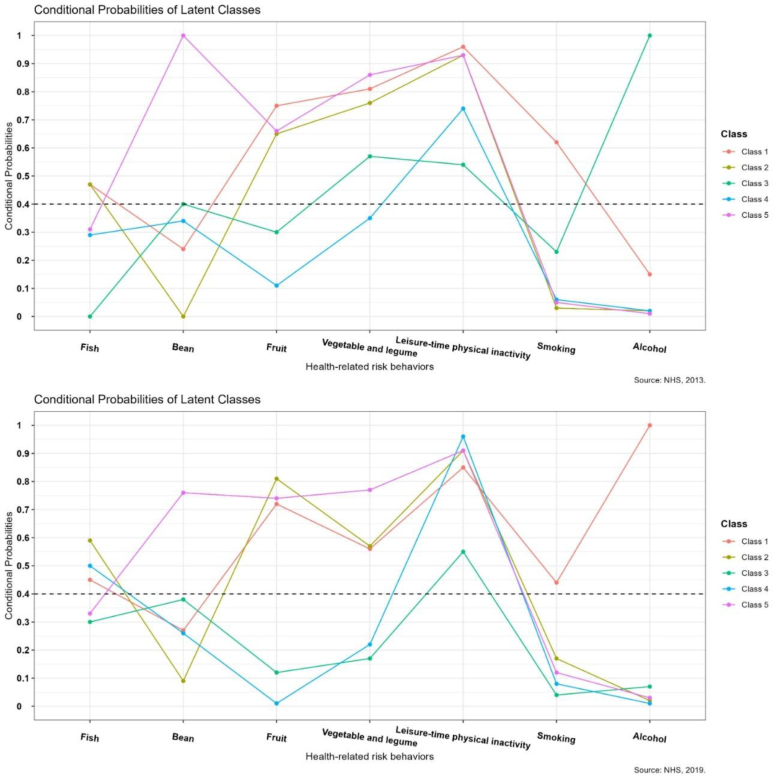
Conditional probabilities of behaviors related to health risk factors among older adults aged ≥60 years interviewed in the National Health Survey, 2013 and 2019, Brazil.

Source: Brazil, PNS 2013 and 2019.

The latent classes identified in 2013 and 2019 were named according to the item-response probabilities, using a conditional probability of ≥0.4 as the criterion for characterizing each class, as detailed in Figure 1. In 2013, five latent classes were identified: *Smoking, Inactivity, and Poor Diet* (smokers, no excessive alcohol consumption, physically inactive, and poor dietary intake, characterized by no consumption of legumes, vegetables, fruits, or fish, but consumption of beans); *Inactivity and Poor Diet with Bean Consumption* (non-smokers, no excessive alcohol consumption, physically inactive, and poor dietary intake characterized by no consumption of legumes, vegetables, fruits, or fish, but consumption of beans); *Alcohol, Inactivity, and Healthy Diet without Vegetable and Legume Consumption* (non-smokers, excessive alcohol consumption, physically inactive, and no consumption of vegetables or legumes; the diet included fruits, fish, and beans); *Inactivity and Healthy Diet* (non-smokers, no excessive alcohol consumption, physically inactive, and a diet including legumes, vegetables, fruits, fish, and beans); and *Inactivity and Poor Diet with Fish Consumption* (non-smokers, no excessive alcohol consumption, physically inactive, and no consumption of legumes, vegetables, fruits, or beans, but consumption of fish).

In 2019, five latent classes were also identified: *Smoking, Alcohol Consumption, Physical Inactivity, and Unhealthy Diet* (smokers, consume alcohol, do not consume vegetables, legumes, or fish, but consume beans); *Physical Inactivity and Unhealthy Diet with Bean Consumption* (non-smokers, do not consume alcohol, do not consume vegetables, legumes, fruits, or fish, but consume beans); *Physical Inactivity and Healthy Diet* (non-smokers, do not consume alcohol, physically inactive, and follow a healthy diet including vegetables, legumes, fruits, beans, and fish); *Physical Inactivity and Healthy Diet Without Fish Consumption* (non-smokers, do not consume alcohol, physically inactive, consume vegetables, legumes, fruits, and beans, but not fish); and *Physical Inactivity and Unhealthy Diet with Fish Consumption* (non-smokers, do not consume alcohol, physically inactive, do not consume vegetables, legumes, fruits, or beans, but consume fish).

Chart 1 presents the distribution of older adults across latent classes estimated separately for 2013 and 2019. In 2013, Class 1, "Smoking, Inactivity, and Poor Diet," accounted for 10.2% (95%CI, 9.2–11.2), whereas in 2019, a class with similar characteristics had a prevalence of 14.1% (95%CI, 13.4–14.8). Class 2, "Inactivity and Poor Diet with Bean Consumption," the most prevalent class in 2013, accounted for 40.2% (95%CI, 38.6–41.8), while in 2019, a class with a similar profile showed a lower proportion (27.4%; 95%CI, 26.4–28.4).

**Chart 1:** Distribution of older adults by latent classes in the National Health Survey for the years 2013 and 2019, with estimates of proportions and confidence intervals.

Class	PNS 2013		PNS 2019	
	Class name	% (95%CI)	Class name	% (95%CI)
1	Smoking, physical inactivity, and inadequate diet	10.2 (9.2–11.2)	Smoking, alcohol consumption, physical inactivity, and inadequate diet	14.1 (13.4–14.8)
2	Physical inactivity and inadequate diet with bean consumption	40.2 (38.6–41.8)	Physical inactivity and inadequate diet with bean consumption	27.4 (26.4–28.4)
3	Alcohol consumption, physical inactivity, and healthy diet without vegetable and legume consumption	1.2 (0.90–1.6)	Physical inactivity and healthy diet	3.3 (2.9–3.7)
4	Physical inactivity and healthy diet	31.2 (29.6–32.8)	Physical inactivity and healthy diet without fish consumption	33.4 (32.3–34.5)
5	Physical inactivity and inadequate diet with fish consumption	17.2 (16.1–18.3)	Physical inactivity and inadequate diet with fish consumption	21.8 (20.9–22.8)

PNS: National Health Survey (*Pesquisa Nacional de Saúde*); CI: confidence interval.

Source: Brazil, PNS 2013 and 2019.

Class 3 showed more substantial changes: in 2013, it was characterized by "Alcohol, Inactivity, and Healthy Diet without Vegetable and Legume Consumption" (1.2%; 95%CI: 0.9–1.6), whereas in 2019, a class with a distinct configuration (Inactivity and Healthy Diet) emerged, with a prevalence of 3.3% (95%CI 2.9–3.7). Similarly, Class 4 maintained a predominance of a healthy diet associated with physical inactivity; however, in 2019, it did not include fish consumption, showing a prevalence of 33.4% (95%CI 32.3–34.5), compared with 31.2% (95%CI 29.6–32.8) in 2013. Finally, Class 5, "Inactivity and Poor Diet with Fish Consumption," increased from 17.2% (95%CI 16.1–18.3) in 2013 to 21.8% (95%CI 20.9–22.8) in 2019 (Chart 1).

The 2013 multinomial regression indicated an association between socioeconomic and demographic variables and the latent classes identified in the two PNS waves. In 2013, the reference class was Class 1 (Smoking, Inactivity, and Poor Diet). For Class 2 (Inactivity and Poor Diet with Bean Consumption), Black and Brown individuals showed lower odds of belonging to this class compared with White individuals (OR 0.62; 95%CI 0.49–0.77 and OR 0.77; 95%CI 0.66–0.90, respectively). Conversely, women had significantly higher odds (OR 2.59; 95%CI 2.23–3.00; p<0.0001), as did older adults aged ≥65 years, particularly those aged ≥75 years (OR 2.91; 95%CI 2.38–3.56). Individuals without a spouse showed lower odds of belonging to this class compared with the reference class (OR 0.66; 95%CI 0.57–0.76). For Class 3 (Alcohol, Inactivity, and Healthy Diet excluding Vegetables and Legumes), Black individuals showed higher odds (OR 2.23; 95%CI 1.26–3.94), whereas women were less likely to belong to this class (OR 0.61; 95%CI 0.39–0.95). High school education (OR 3.99; 95%CI 2.30–6.92) and higher education (OR 5.83; 95%CI 3.14–10.83) were also associated with this class. Class 4 (Inactivity and Healthy Diet) was associated with female sex (OR 4.24; 95%CI 3.61–4.97) and older age groups compared with the reference class, whereas individuals without a spouse (OR 0.69; 95%CI 0.59–0.80) had lower odds compared with those with a spouse. Regarding Class 5 (Inactivity and Poor Diet with Fish Consumption), women were also more likely to belong to this class (OR 3.29; 95%CI 2.80–3.86), whereas Black race was negatively associated with this class (OR 0.72; 95%CI 0.56–0.93) (Table 3).

**Table 3:** Multinomial logistic regression of the association between latent classes and socioeconomic and demographic variables among older adults in the National Health Survey for the years 2013 and 2019.

Characteristic		2013				2019			
		Class 2 OR (95%CI)	Class 3 OR (95%CI)	Class 4 OR (95%CI)	Class 5 OR (95%CI)	Class 2 OR (95%CI)	Class 3 OR (95%CI)	Class 4 OR (95%CI)	Class 5 OR (95%CI)
Race/skin color									
	White	ref	ref	ref	ref	ref	ref	ref	ref
	Black	0.62 (0.49–0.77)*	2.23 (1.26–3.94)*	0.59 (0.46–0.76)*	0.72 (0.56–0.93)	0.80 (0.62–1.01)	0.51 (0.40–0.66)*	0.49 (0.38–0.62)*	0.72 (0.56–0.92)*
	Brown	0.77 (0.66–0.90)*	1.43 (0.95–2.17)	0.64 (0.55–0.76)*	0.86 (0.73–1.02)	0.89 (0.75–1.05)	0.62 (0.52–0.74)*	0.56 (0.47–0.66)*	0.93 (0.78–1.11)
	Others	0.43 (0.24–0.79)*	1.06 (0.23–4.86)	0.86 (0.48–1.54)	1.01 (0.56–1.83)	0.59 (0.35–0.98)*	0.57 (0.34–0.97)	0.44 (0.26–0.74)*	0.73 (0.43–1.24)
Sex									
	Male	ref	ref	ref	ref	ref	ref	ref	ref
	Female	2.59 (2.23–3.00)*	0.61 (0.39-0.95)*	4.24 (3.61–4.97)*	3.29 (2.80–3.86)*	4.28 (3.51–5.22)*	5.64 (4.61–6.90)*	8.08 (6.62–9.86)*	6.43 (5.25–7.88)*
Age range									
	60–64	ref	ref	ref	ref	ref	ref	ref	ref
	65–69	1.40 (1.18–1.67)*	0.98 (0.64–1.50)	1.55 (1.29–1.87)*	1.47 (1.22–1.79)*	1.18 (0.99–1.40)	1.44 (1.20–1.72)*	1.46 (1.22–1.74)*	1.44 (1.20–1.73)*
	70–74	1.73 (1.42–2.11)*	0.64 (0.36–1.16)	1.84 (1.49–2.27)*	1.77 (1.43–2.20)*	2.21 (1.74–2.81)*	2.77 (2.17–3.54)*	3.16 (2.48–4.02)*	2.62 (2.04–3.35)*
	75 or more	2.91 (2.38–3.56)*	0.60 (0.30–1.21)	3.15 (2.54–3.91)*	3.54 (2.85–4.39)*	3.88 (2.97–5.07)*	4.42 (3.37–5.81)*	7.25 (5.55–9.47)*	5.41 (4.12–7.10)*
Marital status									
	With spouse/partner	ref	ref	ref	ref	ref	ref	ref	ref
	Without spouse/partner	0.66 (0.57–0.76)*	0.88 (0.61–1.28)	0.69 (0.59–0.80)*	0.83 (0.71–0.97)*	0.74 (0.63–0.86)*	0.77 (0.65–0.90)*	0.66 (0.56–0.77)*	0.98 (0.83–1.15)
Education									
	Incomplete primary education	ref	ref	ref	ref	ref	ref	ref	ref
	Incomplete secondary education	1.20 (0.92–1.57)	3.22 (1.77–5.84)*	1.83 (1.39–2.41)*	1.13 (0.85–1.52)	0.64 (0.49–0.83)*	1.63 (1.25–2.13)*	1.07 (0.82–1.39)	0.81 (0.61–1.07)
	Incomplete higher education	1.39 (1.06–1.81)*	3.99 (2.30–6.92)*	2.81 (2.15–3.67)*	1.77 (1.33–2.34)*	0.40 (0.33–0.50)*	1.82 (1.48–2.24)*	0.90 (0.73–1.11)	0.63 (0.51–0.79)*
	Complete higher education	1.16 (0.81–1.66)	5.83 (3.14–10.83)*	3.23 (2.28–4.58)*	1.75 (1.21–2.53)*	0.34 (0.25–0.48)*	2.09 (1.55–2.82)*	0.83 (0.62–1.13)	0.87 (0.64–1.19)
Religion									
	With religion	ref	ref	ref	ref	ref	ref	ref	ref
	Without religion	0.45 (0.39–0.53)*	0.52 (0.36–0.76)*	0.39 (0.33–0.46)*	0.58 (0.49–0.68)*	0.56 (0.47–0.66)*	0.32 (0.27–0.38)*	0.44 (0.37–0.52)*	0.59 (0.50–0.70)*
Income									
	Up to ½ MW	ref	ref	ref	ref	ref	ref	ref	ref
	1/2 to 1 MW	1.05 (0.85–1.29)	1.04 (0.45–2.42)	1.11 (0.88–1.42)	0.89 (0.71–1.12)	0.86 (0.66–1.12)	1.11 (0.83–1.48)	0.95 (0.73–1.25)	0.76 (0.58–1.00)*
	1 to 2 MW	1.32 (1.06–1.64)*	2.06 (0.92–4.63)	2.39 (1.86–3.07)*	1.11 (0.87–1. 41)	0.60 (0.46–0.78)*	1.22 (0.91–1.62)	0.97 (0.74–1.28)	0.56 (0.43–0.74)*
	2 to 3 MW	1.09 (0.80–1.48)	3.73 (1.56–8.94)*	2.75 (1.99–3.80)*	1.16 (0.83–1.60)	0.50 (0.35–0.70)*	1.67 (1.18–2.38)*	1.09 (0.78–1.53)	0.55 (0.39–0.78)*
	More than 3 MW	1.02 (0.73–1.40)	6.23 (2.66–14.58)*	3.91 (2.80–5.47)*	1.40 (1.00–1.97)	0.38 (0.27–0.53)*	2.22 (1.57–3.13)*	0.98 (0.70–1.37)	0.51 (0.36–0.72)*
Area type									
	Urban	ref	ref	ref	ref	ref	ref	ref	ref
	Rural	0.46 (0.38–0.55)*	0.68 (0.43–1.06)	1.13 (0.96–1.33)	0.71 (0.62–0.81)	1.53 (1.27–1.83)	0.68 (0.56–0.83)*	0.98 (0.81–1.17)	1.34 (1.11–1.62)

* Statistically significant associations (p<0.05).

OR: odds ratio; CI: confidence interval; MW: minimu wage, ref: reference category.

Source: Brazil, PNS 2013 and 2019.

In 2019, associations were also observed between socioeconomic and demographic variables and the latent classes. Class 1 served as the reference (Smoking, Alcohol, Inactivity, and Poor Diet). In Class 2, Black (OR 0.80; 95%CI 0.62–1.01), Brown (OR 0.89; 95%CI 0.75–1.05), and other racial groups (OR 0.59; 95%CI 0.35–0.98) continued to show lower odds, whereas women and older adults aged ≥75 years showed higher odds. In Class 3 (Inactivity and Healthy Diet), Black, Brown, and other racial groups showed lower odds, whereas women (OR 5.64; 95%CI 4.61–6.90) and older adults, particularly those aged ≥70 years, showed higher odds. Class 4 (Inactivity and Healthy Diet without Fish Consumption) showed lower odds of membership among Black, Brown, and other racial groups, whereas women (OR 8.08; 95%CI 6.62–9.86) and older adults, particularly those aged ≥75 years, showed higher odds of belonging to this class. Finally, in Class 5 (Inactivity and Poor Diet), women showed higher odds (OR 6.43; 95%CI 5.25–7.88), as did older adults aged ≥75 years, whereas the Black racial group showed lower odds of membership.

## DISCUSSION

Behavioral patterns exert a significant influence on overall health status. Smoking, alcohol consumption, physical inactivity, and dietary irregularities reflect habits and lifestyles associated with risk factors^17^.

In the first year, patterns of smoking, physical inactivity, and dietary variety stood out; in the second, higher alcohol consumption and lower fish intake were observed in some groups. Findings from other authors corroborate the high prevalence of modifiable risk factors in Brazil^18^.

Stability was observed in the age and sex distribution, whereas race, education, and income showed changes over the period. Women were more likely to belong to the physical inactivity classes, whether characterized by poor or healthy diets, consistent with the literature indicating higher levels of inactivity among older women due to family caregiving responsibilities and physical limitations^19^. Conversely, they were more represented in the healthy diet classes and less likely to belong to the alcohol consumption class, consistent with existing literature^19^.

Marital status influenced adherence to healthy eating habits: older adults without a spouse were less likely to belong to the healthy-diet classes. These findings may stem from a lack of social and family support^20^, which can compromise well-being and hinder the adoption of healthy behaviors (such as a balanced diet and regular physical activity). Another finding was the selective association between higher education levels and health behaviors. Individuals with higher levels of education were more likely to belong to classes characterized by alcohol consumption — a finding consistent with the literature — but were also more likely to belong to the healthy-diet classes. Despite these mixed results, higher education facilitates access to healthcare and healthier living conditions^21,22^.

Racial inequalities in health behaviors were observed, highlighting socioeconomic disadvantages and disparities in access to healthy foods^23–25^. In 2013, Black individuals showed a higher likelihood of belonging to the "Alcohol, Inactivity, and Healthy Diet without Vegetable and Legume Consumption" class. In 2019, Black and Brown individuals were less likely to belong to the classes characterized by a healthy diet.

An increase was observed in the lowest income quintiles, indicating the persistence of socioeconomic inequalities that may affect the prevalence of NCDs and their risk factors^24^. In this context, older adults were also more likely to belong to categories of physical inactivity, possibly related to functional decline. Evidence indicates that physical activity contributes to longer life expectancy and reductions in NCDs and disabilities^24^.

Opposite trends were observed in the prevalence of behaviors related to the assessed risk factors. On the one hand, adherence to positive health behaviors (consumption of fruits and vegetables and physical activity) increased, which may reflect public health policies promoting healthy eating and physical activity^24,25^. On the other hand, worsening trends in negative behaviors (excessive alcohol consumption and a decline in fish consumption) were observed, alongside stability in smoking rates and bean consumption. These changes align with previous studies describing improvements in estimates of positive health behaviors and worsening trends in negative ones among the general Brazilian population over time^18,23^.

Multinomial regression identified significant associations between socioeconomic and demographic factors and latent classes. In both years, Black and Brown individuals were less likely to belong to the "Inactivity and Inadequate Diet with Bean Consumption" class, corroborating evidence regarding inequalities in access to health and food resources^26,27^.

In both samples, the absence of physical activity was observed across all groups. The WHO recommends that older adults (aged ≥60 years) engage in 150 minutes of moderate-intensity physical activity or 75 minutes of vigorous-intensity physical activity per week to maintain good health and prevent NCDs^27^.

A latent class grouped the characteristics of smoking, physical inactivity, and an unhealthy diet. Smoking is a major modifiable risk factor that negatively affects health across various stages of life^28^. It is associated with other harmful habits (unbalanced diet, a sedentary lifestyle, and alcohol consumption) that increase the risk of NCDs^29^. This combination predisposes individuals to significant changes in physical and emotional health, resulting in personal and family suffering, as well as high social costs, making it a key focus for public health interventions^28^.

In both years, classes characterized by healthy eating, associated with physical inactivity and the absence of smoking and alcohol consumption, were identified. According to the WHO, healthy dietary practices adopted early in life yield long-lasting health benefits for individuals^30^.

Healthy food consumption is determined by socioeconomic and demographic factors. Brazil is among the most unequal countries and exhibits high levels of malnutrition in the general population^31^. A study using PNS data indicates higher consumption of healthy foods among individuals with higher income and education levels, a group that also showed a higher prevalence of excessive alcohol consumption, highlighting the coexistence of protective and risk-related behaviors^5^.

Thus, the clustering of modifiable health risk factors reflects a complex interplay of biopsychosocial and racial factors. The co-occurrence of these factors suggests that health promotion interventions must be multidimensional rather than address risk factors in isolation. Consequently, strategies targeting synergistic health behaviors must take into account the social determinants that are fundamental to the adoption and maintenance of healthy behaviors.

The findings reinforce the importance of adopting and maintaining healthy behaviors, indicating that risk patterns result from a combination of lifestyle habits. These patterns influence healthcare, social engagement, access to interventions and services, and the interaction between individuals and the environment. International evidence supports this perspective. A study conducted in Thailand identified poor dietary patterns and overweight/obesity as key risk factors for NCDs, highlighting the need for actions aimed at reducing inequities^32^. In Africa, NCDs and mental health were found to be associated with the interplay of environmental, behavioral, biological, and social factors^33^. Parallels with the Brazilian context include the persistence of inequities and structural barriers that limit access to health services, medications, and technologies for NCD management.

In Brazil, public policies such as the National Health Policy for the Elderly, the Guide to Care for the Elderly, the Statute of the Elderly, and the Strategic Action Plan for Tackling NCDs provide important foundations for promoting healthy aging and reducing inequalities. The National Health Surveillance Policy reinforces the centrality of territory and equity in the care of vulnerable populations, elements directly related to the findings of this study^34^.

Finally, methodological limitations should be highlighted. The behaviors analyzed do not cover the full spectrum of lifestyle; the data are self-reported and subject to bias; and LCA is exploratory, dependent on variable selection, and does not allow causal inferences. Methodological changes between editions of the PNS may also have influenced the observed prevalence rates.

Despite these limitations, the results contribute to the planning of care, surveillance, and health promotion actions, providing a basis for interventions aimed at reducing combined risk behaviors and promoting active and healthy aging.
