# Supplementary material for: Patterns of behaviors related to modifiable health risk factors among Brazilian older adults: Data from the 2013 and 2019 National Health Surveys
Source: Rev Bras Epidemiol. 2026 Aug 17;29:e260042. doi: 10.1590/1980-549720260042 (PMC13480969; doi:10.1590/1980-549720260042)
Supplement: Translation

# Translation: Padrões de comportamentos relacionados aos fatores de risco à saúde modificáveis em pessoas idosas brasileiras: dados da Pesquisa Nacional de Saúde 2013 e 2019

**DOI:** 10.1590/1980-549720260042.2

**Authors:** Mariana Sousa de Abreu Menezes, Zayanna Christine Lopes Lindôso, Ana Karina Teixeira da Cunha França, Poliana Cristina de Almeida Fonseca Viola, Bruno Luciano Carneiro Alves de Oliveira

**Affiliations:** I Universidade Federal do Maranhão, Programa de Pós-Graduação em Saúde Coletiva – São Luís (MA), Brasil.; II Universidade Federal de Pelotas, Colegiado do Curso de Terapia Ocupacional – Pelotas (RS), Brasil.

**Keywords:** Pessoas idosas, Envelhecimento saudável, Determinantes sociais da saúde, Análise de classes latentes, Comportamentos de riscos combinados

## Abstract

**Objetivo:**

Analisar os padrões de comportamentos de risco à saúde modificáveis em pessoas idosas brasileiras (≥60 anos) utilizando dados da Pesquisa Nacional de Saúde 2013 e 2019.

**Métodos:**

Estudo transversal com base nos dados secundários provenientes da Pesquisa Nacional de Saúde de 2013 e 2019. Os padrões de comportamento foram definidos pela Análise de Classes Latentes de acordo com quatro domínios principais: tabagismo, consumo de álcool, atividade física e alimentação. Foram calculadas as frequências relativas e seus Intervalos de Confiança de 95% (IC95%) para as variáveis de estilo de vida, bem como para as variáveis independentes associadas a fatores socioeconômicos e demográficos.

**Resultados:**

Foram identificados cinco padrões de comportamento em cada ano. Em 2013, identificaram-se padrões relacionados a hábitos não saudáveis (fumo), inatividade física e padrões alimentares (incluindo consumo de feijão, vegetais, legumes e peixes). Em 2019, os achados também envolveram os mesmos padrões encontrados em 2013, com o diferencial de que o consumo de peixe apresentou maior relevância na diferenciação entre classes, embora sua prevalência populacional tenha diminuído no período. A inatividade física foi associada a hábitos prejudiciais: alimentação inadequada, tabagismo e consumo de álcool. Idade, sexo, escolaridade e raça influenciaram significativamente esses padrões.

**Conclusão:**

No Brasil, o envelhecimento não saudável sobrepõe-se a múltiplos fatores de risco modificáveis. A compreensão desses padrões e seus determinantes permite elaborar estratégias que reduzam desigualdades em saúde e promovam qualidade de vida e bem-estar entre pessoas idosas. A abordagem combinada de fatores de risco contribui para políticas públicas mais efetivas.

## INTRODUÇÃO

Em 2022, o Brasil possuía cerca de 32 milhões de pessoas com 60 anos ou mais, o que representa 15,8% da população^1^. Projeções indicam que até 2030 o número de idosos superará o de crianças e adolescentes no país^2^. Contudo, proporção significativa dessa população, em 2023, assumia comportamentos prejudiciais à saúde: 56% relataram sedentarismo; 14%, uso abusivo de bebidas alcoólicas; 12% fumavam; e apenas 18% relataram consumo alimentar adequado (frutas e verduras)^1–4^.

Tais comportamentos estão associados às Doenças Crônicas Não Transmissíveis (DCNT), que são as principais causas de morbimortalidade no mundo e afetam de modo mais relevante as pessoas idosas. A prevalência de DCNT é influenciada por um conjunto de fatores de risco à saúde modificáveis^5,6^. Entretanto, o papel dos comportamentos de risco à saúde ainda tende a ser analisado de forma isolada, ignorando a interdependência deles, sua coocorrência e sobreposição na formação de específicos padrões de saúde^7,8^. Padrões de comportamentos de risco amplificam seus efeitos negativos, pois sinergicamente comprometem a qualidade de vida, agravam o estado de saúde geral, reduzem as chances de longevidade e aumentam o risco de mortalidade prematura^9,10^.

Pessoas idosas geralmente apresentam padrões de comportamento de risco à saúde mais complicados e modelados por determinantes sociais de saúde^11^. Esses combinados padrões de comportamentos refletem hábitos (saudáveis ou não) adotados ao longo do ciclo de vida, que são geralmente vivenciados em conjunto e mais difíceis de serem modificados individualmente.

Dessa forma, o monitoramento dos comportamentos relacionados aos fatores de risco à saúde e de seus determinantes socioeconômicos, geográficos e raciais pode contribuir com maior compreensão dos efeitos de estilos de vida, gestão de DCNT e qualidade de intervenções multidisciplinares em pessoas idosas. Logo, inquéritos de saúde como os da Pesquisa Nacional de Saúde (PNS) 2013 e 2019 permitem tais análises no contexto brasileiro^12^.

Diante disso, este estudo buscou analisar os padrões de comportamentos relacionados aos fatores de risco à saúde modificáveis em pessoas idosas brasileiras nos anos de 2013 e 2019.

## MÉTODOS

### Tipo e local de estudo

Trata-se de um estudo transversal com base nos dados secundários provenientes da PNS 2013 e 2019. A PNS é um inquérito transversal, de base populacional, representativo do Brasil e com abordagem domiciliar realizado em conjunto pelo Instituto Brasileiro de Geografia e Estatística (IBGE) e pelo Ministério da Saúde (MS).

### Contexto

A PNS busca apresentar resultados representativos da população brasileira. Para isso, utiliza um desenho de amostragem complexa^13^, organizada por conglomerados, em três estágios de seleção: setores censitários (unidades primárias), domicílios (unidades secundárias) e moradores adultos com 18 anos ou mais (unidades terciárias). Todavia, em 2019, no terceiro estágio, o morador tinha 15 anos ou mais de idade^14,15^.

A PNS 2013 possuiu desenho amostral com entrevistas em 6.062 Unidades Primárias de Amostragem (UPA) e 64.348 domicílios^12^; e a PNS de 2019, 8.031 UPA e 94.114 domicílios. Para o presente estudo, foram analisadas as respostas do questionário individual em pessoas idosas (≥60 anos). A amostra final analisada incluiu 11.177 idosos da PNS 2013 e 22.728 da PNS 2019.

### Variáveis

As variáveis relacionadas ao estilo de vida foram organizadas em quatro domínios: tabagismo, consumo de álcool, prática de atividade física e alimentação, identificadas no Módulo P do questionário do morador selecionado.

A alimentação incluiu o consumo semanal de feijão, frutas, verduras ou legumes e peixe, mensurados em número de vezes por semana. Essas variáveis foram dicotomizadas conforme recomendações de diretrizes nacionais e internacionais, sendo considerado consumo adequado ≥5 vezes por semana para feijão, frutas e verduras/legumes, e ≥2 vezes por semana para peixe. O consumo de álcool foi definido de acordo com o número de doses ingeridas em uma única ocasião nos últimos 30 dias, categorizado como consumo abusivo quando ≥5 doses para homens ou ≥4 doses para mulheres.

Quanto à prática de atividade física no lazer, classificaram-se como fisicamente ativos os indivíduos que atingiram pelo menos 150 minutos semanais de atividade moderada ou 75 minutos de atividade vigorosa, de acordo com as recomendações da Organização Mundial da Saúde (OMS). O tabagismo foi definido considerando fumantes aqueles que relataram uso atual de produtos de tabaco, diário ou ocasional.

As variáveis independentes incluíram: sexo, idade (anos), cor ou raça, viver com companheiro, religião, escolaridade, renda e área de moradia.

### Análise estatística

Na análise descritiva, foram calculadas as frequências relativas e seus Intervalos de Confiança de 95% (IC95%) para os comportamentos de saúde e variáveis socioeconômicas e demográficas.

Para definição dos padrões de comportamentos, foi realizada a Análise de Classes Latentes (LCA), método estatístico que identifica grupos distintos com base nos padrões de respostas observados em variáveis categóricas^16^. A LCA foi utilizada para identificar padrões de comportamento relacionados a fatores de risco modificáveis, com base nas variáveis categóricas de estilo de vida^16^, estimadas separadamente para os anos de 2013 e 2019. Na construção da variável latente, foram elaborados e avaliados modelos com diversos números de classes latentes até determinar o modelo mais apropriado para descrevê-la. A seleção do modelo ideal foi guiada pelos Critérios de Informação de Akaike (AIC), Informação Bayesiano (BIC), Informação Bayesiano Ajustado (aBIC) e log-verossimilhança, com preferência aos modelos que apresentaram os menores valores desses critérios em comparação com os modelos anteriores^16^; também foi considerado o maior valor da entropia. A decisão final sobre o número de classes levou em conta a interpretabilidade e a coerência dos padrões identificados.

Para testar a associação entre as classes latentes e as variáveis socioeconômicas e demográficas independentes, foram estimados modelos de regressão logística multinomial ajustada. A medida de efeito utilizada foi a *Odds Ratio* (OR), com seus respectivos IC95% e nível de significância de p<0,05. Para realização das análises estatísticas, foi considerado o desenho complexo da amostra, estratos e UPA. As análises foram realizadas no software *RStudio*, versão 2023.9.0.463, e na análise de classe latente utilizou-se o pacote "poLCA".

A PNS de 2013 e 2019 foi aprovada pela Comissão Nacional de Ética em Pesquisa para Seres Humanos do Ministério da Saúde. Para a edição de 2013, o parecer foi o n^o^ 328.159, e para 2019, n^o^ 3.529.376.

#### Declaração de disponibilidade de dados:

Todo o conjunto de dados que dá suporte aos resultados deste estudo está disponível mediante solicitação ao autor correspondente. O conjunto de dados não está publicamente disponível devido à integração de bancos de dados realizados pelos autores.

## RESULTADOS

O estudo teve amostra de 11.177 indivíduos ≥60 anos entrevistados pela PNS 2013 e 22.728 pela PNS 2019.

A Tabela 1 apresenta as características socioeconômicas e demográficas em 2013 e 2019. Não se observaram mudanças na distribuição por sexo. Mulheres foram maioria em 2013 (56,94%; IC95% 54,8–58,0) e 2019 (56,7%; IC95% 55,6–57,8). A proporção de brancos diminuiu entre os anos (2013: 53,8%; IC95% 51,9–55,6; 2019: 50,5%; IC95% 49,3–51,8; p=0,02). Houve mudança significativa na escolaridade: o ensino fundamental incompleto caiu de 70,7% (IC95% 68,9–72,4) para 63,3% (IC95% 62,0–64,5), enquanto o nível superior completo aumentou de 9,4% (IC95% 8,1–10,7) para 11,3% (IC95% 10,5–12,1; p<0,001). A distribuição de renda diferiu: aumentou no primeiro quintil (2013: 20,6%; IC95% 19,2–22,0; 2019: 24,4%; IC95% 23,4–25,4; p<0,001). Quanto à área de residência, a urbana prevaleceu, mas sem mudanças significativas.

**Tabela 1:** Características socioeconômicas e demográficas de pessoas idosas entrevistadas pela Pesquisa Nacional de Saúde, anos de 2013 e 2019 no Brasil.

Variáveis		IC95%		
		2013 (n=11.177)	2019 (n=22.728)	p-valor
Sexo				
	Masculino	43,6 (42,0–45,2)	43,3 (42,2–44,4)	0,76
	Feminino	56,4 (54,8–58,0)	56,7 (55,6–57,8)	
Faixa etária				
	60 a 64	32,0 (30,5–33,5)	31,1 (30,0–32,1)	0,72
	65 a 69	24,6 (23,3–25,9)	25,2 (24,3–26,1)	
	70 a 74	18,3 (17,0–19,5)	18,1 (17,3–18,9)	
	75 ou mais	25,2 (23,8–26,5)	25,6 (24,6–26,5)	
Raça				
	Branca	53,8 (51,9–55,6)	50,5 (49,3–51,8)	0,02
	Preta	9,2 (8,2–10,2)	10,3 (9,6–11.0)	
	Parda	35,6 (33,9–37,2)	37,4 (36,2–38,5)	
	Outros	1,4 (1,1–1,8)	1,8 (1,5–2,1)	
Estado civil				
	Com cônjuge	53,5 (51,9–55,1)	50,7 (49,5–51,8)	0,004
	Sem cônjuge	46,5 (44,9–48,1)	49,3 (48,2–50,5)	
Escolaridade				
	Até fundamental incompleto ou equivalente	70,7 (68,9–72,4)	63,3 (62,0–64,5)	0,001
	Médio incompleto ou equivalente	8,1 (7,2–9,0)	9,5 (8,9–10,2)	
	Superior incompleto ou equivalente	11,8 (10,8–12,9)	15,9 (15,0–16,8)	
	Superior completo	9,4 (8,1–10,7)	11,3 (10,5–12,1)	
Renda				
	1° Quintil	20,6 (19,2–22,0)	24,4 (23,4–25,4)	0,001
	2° Quintil	22,7 (21,3–24,1)	17,4 (16,6–18,2)	
	3° Quintil	16,6 (15,3–18,0)	16,1 (15,2–16,9)	
	4° Quintil	21,3 (19,9–22,7)	21,8 (20,8–22,7)	
	5° Quintil	18,7 (17,1–20,2)	20,4 (19,3–21,5)	
Religião				
	Sim	50,1 (48,3–51,8)	48,5 (47,4–49,7)	0,15
	Não	49,9 (48,2–51,7)	51,5 (50,3–52,6)	
Área de moradia				
	Urbana	85,2 (83, 7–86,6)	85,5 (84,6–86,3)	0,76
	Rural	14,8 (13,4–16,3)	14,5 (13,7–15,4)	

Fonte: Brasil, PNS 2013 e 2019.

Entre os anos, a prevalência dos fatores de risco à saúde modificáveis foi estimada (dados não mostrados). Tabagismo (2013: 12,2%; IC95% 11,1–13,2; 2019: 11,1%; IC95% 10,5–11,8) e consumo de feijão (2013: 71,4%; IC95% 69,9–72,9; 2019: 70,2%; IC95% 69,1–71,2) não apresentaram mudança estatisticamente significante (p>0,05). O consumo excessivo de álcool aumentou de 4,2% (IC95% 3,6–4,9) para 5,8% (IC95% 5,2–6,3; p<0,001). A atividade física no lazer aumentou significativamente: de 13,4% (IC95% 12,3–14,5) para 19,4% (IC95% 18,5–20,4; p<0,001). O consumo de vegetais e legumes (de 38,1% [IC95% 36,3–39,9] para 63,4% [62,3–64,5; p<0,001]) e de frutas (de 55% [IC95% 53,3–56,7] para 59,2% [IC95% 58,1–60,3; p<0,001]) também aumentou significativamente. Já o consumo de peixe diminuiu: 58,2% (IC95% 56,3–60,1) para 50% (IC95% 48,8–51,2; p<0,001).

A Tabela 2 apresenta as estatísticas de ajuste utilizadas para determinar o número ideal de classes latentes. A seleção do número de classes considerou critérios estatísticos (AIC, BIC, aBIC, log-verossimilhança e entropia), bem como a interpretabilidade substantiva das classes. Observou-se redução dos valores de AIC, BIC e aBIC até o modelo de cinco classes, com estabilidade nos modelos subsequentes. A escolha do modelo final considerou o equilíbrio entre ajuste estatístico, parcimônia e entropia, priorizando soluções interpretáveis e teoricamente plausíveis.

**Tabela 2:** Critérios para a seleção do número de classes latentes formadas por pessoas idosas na Pesquisa Nacional de Saúde para os anos de 2013 e 2019.

Classes	2013							2019						
	Log–verossimilhança	gl dos resíduos	AIC	BIC	aBIC	cAIC	Entropia	Log-verossimilhança	gl dos resíduos	AIC	BIC	aBIC	cAIC	Entropia
2	-39340,91	112	78535,31	78821,65	78773,98	78836,65	0,405	-83557,24	112	167144,5	167264,9	167217,3	167279,9	0,478
3	-39242,70	104	78535,31	78699,80	78626,71	78722,80	0,494	-83296,85	104	166639,7	166824,4	166751,3	166847,4	0,432
4	-39165,64	96	78393,29	78620,26	78521,75	78651,26	0,501	-83190,60	96	166443,2	166692,2	166593,7	166723,2	0,403
5	**-39140,54**	**88**	**78357,52**	**78644,63**	**78520,69**	**78683,63**	**0,625**	**-83130,20**	**88**	**166338,4**	**166651,6**	**166527,7**	**166690,6**	**0,47**
6	-39123,83	80	78341,67	78685,79	78536,43	78732,79	0,491	-83109,40	80	166312,8	166690,3	166540,9	166800,0	0,417
7	-39116,85	72	78342,05	78746,39	78571,61	78801,39	0,483	-83096,63	72	166303,3	166745,0	166570,2	166800,0	0,395
8	-39111,47	64	78345,77	78810,21	78610,00	78873,21	0,508	-83096,63	72	166298,7	166745,0	166604,4	166867,6	0,422

AIC: Critérios de Informação de Akaike; BIC: Informação Bayesiano; aBIC: Informação Bayesiano Ajustado; cAIC: Critério de Informação de Akaike Consistente.

Em negrito o número de classes selecionadas.

Fonte: Brasil, PNS 2013 e 2019.

As classes identificadas em 2013 e 2019 foram denominadas com base nas probabilidades de resposta aos itens, sendo a probabilidade condicional ≥0,4 critério para caracterizar as classes latentes, conforme detalhado na Figura 1. Em 2013, a divisão de classes foi a seguinte: *Fumo, Inatividade e Alimentação Inadequada* (fumantes, não consomem álcool, inativos e têm uma alimentação inadequada, sem a ingestão de legumes, vegetais, frutas e peixes, mas consumo de feijão); *Inatividade e Alimentação Inadequada com Consumo de Feijão* (não fumantes, não consomem álcool, inativos e têm uma dieta sem legumes, vegetais, frutas e peixes, mas com consumo de feijão); *Álcool, Inatividade e Alimentação Saudável sem Consumo de Vegetais e Legumes* (não fumantes, consumo de álcool em excesso, inativos e não consomem vegetais e legumes; incluem frutas, peixes e feijão na dieta); *Inatividade e Alimentação Saudável* (não fumantes, não consomem álcool e inativos. Sua dieta inclui legumes, vegetais, frutas, peixes e feijão; e, por fim, a classe 5, *Inatividade e Alimentação Inadequada com Consumo de Peixe* (não fumantes, não consomem álcool e inativos; ausência de legumes, vegetais, frutas e feijão, e consumo de peixes).

**Figura 1:** Probabilidades condicionais de comportamentos relacionados a fatores de risco à saúde em pessoas idosas ≥60 anos entrevistadas pela Pesquisa Nacional de Saúde, anos de 2013 e 2019, no Brasil.

Fonte: Brasil, PNS 2013 e 2019.

Em 2019, também foram identificadas cinco classes latentes: *Fumo, Álcool, Inatividade e Alimentação Inadequada* (fumantes, consomem álcool, não consomem vegetais, legumes e peixes, mas incluem feijão); *Inatividade e Alimentação Inadequada com Consumo de Feijão* (não fumantes, não consomem álcool, não consomem vegetais, legumes, frutas ou peixes, mas consomem feijão); *Inatividade e Alimentação Saudável* (não fumantes, não consomem álcool, inativos e seguem uma alimentação saudável, que inclui vegetais, legumes, frutas, feijão e peixe); *Inatividade e Alimentação Saudável sem Consumo de Peixe* (não fumantes, não consomem álcool e inativos, consumindo vegetais, legumes, frutas e feijão, mas sem consumo de peixe); e *Inatividade e Alimentação Inadequada com Consumo de Peixe* (não fumantes, não consomem álcool, inativos, sem vegetais, legumes, frutas e feijão, e consumo de peixe).

O Quadro 1 apresenta a distribuição das pessoas idosas nas classes latentes estimadas separadamente para 2013 e 2019. Em 2013, a classe 1, "Fumo, Inatividade e Alimentação Inadequada", representou 10,2% (IC95% 9,2–11,2), enquanto em 2019 uma classe com características semelhantes apresentou prevalência de 14,1% (IC95% 13,4–14,8). A classe 2, "Inatividade e Alimentação Inadequada com Consumo de Feijão", mais prevalente em 2013, correspondeu a 40,2% (IC95% 38,6–41,8), enquanto em 2019 uma classe com perfil próximo apresentou menor proporção (27,4%; IC95% 26,4–28,4).

**Quadro 1:** Distribuição de pessoas idosas por classes latentes na Pesquisa Nacional de Saúde para os anos de 2013 e 2019 com estimativas de proporção e intervalos de confiança.

Classes	PNS 2013		PNS 2019	
	Nome da classe	% (IC95%)	Nome da classe	% (IC95%)
1	Fumo, inatividade e alimentação inadequada	10,2 (9,2–11,2)	Fumo, álcool, inatividade e alimentação inadequada	14,1 (13,4–14,8)
2	Inatividade e alimentação inadequada com consumo de feijão	40,2 (38,6–41,8)	Inatividade e alimentação inadequada com consumo de feijão	27,4 (26,4–28,4)
3	Álcool, inatividade e alimentação saudável sem consumo de vegetais e legumes	1,2 (0,90–1,6)	Inatividade e alimentação saudável	3,3 (2,9–3,7)
4	Inatividade e alimentação saudável	31,2 (29,6–32,8)	Inatividade e alimentação saudável sem consumo de peixe	33,4 (32,3–34,5)
5	Inatividade e alimentação inadequada com consumo de peixe	17,2 (16,1–18,3)	Inatividade e alimentação inadequada com consumo de peixe	21,8 (20,9–22,8)

PNS: Pesquisa Nacional de Saúde; IC: intervalo de confiança.

Fonte: Brasil, PNS 2013 e 2019.

A classe 3 apresentou mudanças mais expressivas: em 2013, caracterizava-se por "Álcool, Inatividade e Alimentação Saudável sem Consumo de Vegetais e Legumes" (1,2%; IC95%: 0,9–1,6), enquanto, em 2019, emergiu uma classe com configuração distinta (Inatividade e Alimentação Saudável), com prevalência de 3,3% (IC95% 2,9–3,7). De forma semelhante, a classe 4 manteve predominância de alimentação saudável associada à inatividade física, mas em 2019 não incluiu o consumo de peixe, apresentando prevalência de 33,4% (IC95% 32,3–34,5), em comparação a 31,2% (IC95% 29,6–32,8) em 2013. Por fim, a classe 5, "Inatividade e Alimentação Inadequada com Consumo de Peixe", passou de 17,2% (IC95% 16,1–18,3) em 2013 para 21,8% (IC95% 20,9–22,8) em 2019 (Quadro 1).

A regressão multinomial de 2013 indicou associação entre variáveis socioeconômicas e demográficas com as classes latentes nos dois anos da PNS. Em 2013, a classe de referência foi a classe 1 (Fumo, Inatividade e Alimentação Inadequada). Para a classe 2 (Inatividade e Alimentação Inadequada com Consumo de Feijão), pretos e pardos apresentaram menor probabilidade de pertencer a essa classe em comparação aos brancos (OR 0,62; IC95% 0,49–0,77 e OR 0,77; IC95% 0,66–0,90, respectivamente). Já mulheres tiveram probabilidade significativamente maior (OR 2,59; IC95% 2,23–3,00; p<0,0001), assim como pessoas idosas de todas as faixas ≥65 anos, especialmente ≥75 anos (OR 2,91; IC95% 2,38–3,56). Indivíduos sem cônjuge apresentaram menor chance de pertencer a essa classe, em comparação à classe de referência (OR 0,66; IC95% 0,57–0,76). Para a classe 3 (Álcool, Inatividade e Alimentação Saudável sem Consumo de Vegetais e Legumes), pretos mostraram probabilidade aumentada (OR 2,23; IC95% 1,26–3,94), enquanto mulheres foram menos propensas (OR 0,61; IC95% 0,39–0,95). A escolaridade ensino médio (OR 3,99; IC95% 2,30–6,92) e ensino superior (5,83; IC95% 3,14–10,83) estiveram associadas. A classe 4 (Inatividade e Alimentação Saudável) apresentou associação com o sexo feminino (OR 4,24; IC95% 3,61–4,97) e com idades mais avançadas, quando comparadas à classe de referência, enquanto aqueles sem cônjuge (OR 0,69; IC95% 0,59–0,80) tiveram chances reduzidas em comparação com pessoas com cônjuge. Já na classe 5 (Inatividade e Alimentação Inadequada com Consumo de Peixe), as mulheres também se mostraram mais propensas a pertencer a essa classe (OR 3,29; IC95% 2,80–3,86), e a raça preta esteve negativamente associada (OR 0,72; IC95% 0,56–0,93) (Tabela 3).

**Tabela 3:** Regressão logística multinomial da associação entre as classes latentes e as variáveis socioeconômicas e demográficas de pessoas idosas na Pesquisa Nacional de Saúde para os anos de 2013 e 2019.

Variável		2013				2019			
		Classe 2 OR (IC95%)	Classe 3 OR (IC95%)	Classe 4 OR (IC95%)	Classe 5 OR (IC95%)	Classe 2 OR (IC95%)	Classe 3 OR (IC95%)	Classe 4 OR (IC95%)	Classe 5 OR (IC95%)
Raça									
	Branca	ref	ref	ref	ref	ref	ref	ref	ref
	Preta	0,62 (0,49–0,77)*	2,23 (1,26–3,94)*	0,59 (0,46–0,76)*	0,72 (0,56–0,93)	0,80 (0,62–1,01)	0,51 (0,40–0,66)*	0,49 (0,38–0,62)*	0,72 (0,56–0,92)*
	Parda	0,77 (0,66–0,90)*	1,43 (0,95–2,17)	0,64 (0,55–0,76)*	0,86 (0,73–1,02)	0,89 (0,75–1,05)	0,62 (0,52–0,74)*	0,56 (0,47–0,66)*	0,93 (0,78–1,11)
	Outros	0,43 (0,24–0,79)*	1,06 (0,23–4,86)	0,86 (0,48–1,54)	1,01 (0,56–1,83)	0,59 (0,35–0,98)*	0,57 (0,34–0,97)	0,44 (0,26–0,74)*	0,73 (0,43–1,24)
Sexo									
	Masculino	ref	ref	ref	ref	ref	ref	ref	ref
	Feminino	2,59 (2,23–3,00)*	0,61 (0,39-0,95)*	4,24 (3,61–4,97)*	3,29 (2,80–3,86)*	4,28 (3,51–5,22)*	5,64 (4,61–6,90)*	8,08 (6,62–9,86)*	6,43 (5,25–7,88)*
Faixa etária									
	60–64	ref	ref	ref	ref	ref	ref	ref	ref
	65–69	1,40 (1,18–1,67)*	0,98 (0,64–1,50)	1,55 (1,29–1,87)*	1,47 (1,22–1,79)*	1,18 (0,99–1,40)	1,44 (1,20–1,72)*	1,46 (1,22–1,74)*	1,44 (1,20–1,73)*
	70–74	1,73 (1,42–2,11)*	0,64 (0,36–1,16)	1,84 (1,49–2,27)*	1,77 (1,43–2,20)*	2,21 (1,74–2,81)*	2,77 (2,17–3,54)*	3,16 (2,48–4,02)*	2,62 (2,04–3,35)*
	75 ou mais	2,91 (2,38–3,56)*	0,60 (0,30–1,21)	3,15 (2,54–3,91)*	3,54 (2,85–4,39)*	3,88 (2,97–5,07)*	4,42 (3,37–5,81)*	7,25 (5,55–9,47)*	5,41 (4,12–7,10)*
Estado civil									
	Com cônjuge	ref	ref	ref	ref	ref	ref	ref	ref
	Sem cônjuge	0,66 (0,57–0,76)*	0,88 (0,61–1,28)	0,69 (0,59–0,80)*	0,83 (0,71–0,97)*	0,74 (0,63–0,86)*	0,77 (0,65–0,90)*	0,66 (0,56–0,77)*	0,98 (0,83–1,15)
Escolaridade									
	Fundamental incompleto	ref	ref	ref	ref	ref	ref	ref	ref
	Médio incompleto	1,20 (0,92–1,57)	3,22 (1,77–5,84)*	1,83 (1,39–2,41)*	1,13 (0,85–1,52)	0,64 (0,49–0,83)*	1,63 (1,25–2,13)*	1,07 (0,82–1,39)	0,81 (0,61–1,07)
	Superior incompleto	1,39 (1,06–1,81)*	3,99 (2,30–6,92)*	2,81 (2,15–3,67)*	1,77 (1,33–2,34)*	0,40 (0,33–0,50)*	1,82 (1,48–2,24)*	0,90 (0,73–1,11)	0,63 (0,51–0,79)*
	Superior completo	1,16 (0,81–1,66)	5,83 (3,14–10,83)*	3,23 (2,28–4,58)*	1,75 (1,21–2,53)*	0,34 (0,25–0,48)*	2,09 (1,55–2,82)*	0,83 (0,62–1,13)	0,87 (0,64–1,19)
Religião									
	Com religião	ref	ref	ref	ref	ref	ref	ref	ref
	Sem religião	0,45 (0,39–0,53)*	0,52 (0,36–0,76)*	0,39 (0,33–0,46)*	0,58 (0,49–0,68)*	0,56 (0,47–0,66)*	0,32 (0,27–0,38)*	0,44 (0,37–0,52)*	0,59 (0,50–0,70)*
Renda									
	Até ½ SM	ref	ref	ref	ref	ref	ref	ref	ref
	1/2 a 1 SM	1,05 (0,85–1,29)	1,04 (0,45–2,42)	1,11 (0,88–1,42)	0,89 (0,71–1,12)	0,86 (0,66–1,12)	1,11 (0,83–1,48)	0,95 (0,73–1,25)	0,76 (0,58–1,00)*
	1 a 2 SM	1,32 (1,06–1,64)*	2,06 (0,92–4,63)	2,39 (1,86–3,07)*	1,11 (0,87–1,41)	0,60 (0,46–0,78)*	1,22 (0,91–1,62)	0,97 (0,74–1,28)	0,56 (0,43–0,74)*
	2 a 3 SM	1,09 (0,80–1,48)	3,73 (1,56–8,94)*	2,75 (1,99–3,80)*	1,16 (0,83–1,60)	0,50 (0,35–0,70)*	1,67 (1,18–2,38)*	1,09 (0,78–1,53)	0,55 (0,39–0,78)*
	Mais de 3 SM	1,02 (0,73–1,40)	6,23 (2,66–14,58)*	3,91 (2,80–5,47)*	1,40 (1,00–1,97)	0,38 (0,27–0,53)*	2,22 (1,57–3,13)*	0,98 (0,70–1,37)	0,51 (0,36–0,72)*
Tipo de área									
	Urbana	ref	ref	ref	ref	ref	ref	ref	ref
	Rural	0,46 (0,38–0,55)*	0,68 (0,43–1,06)	1,13 (0,96–1,33)	0,71 (0,62–0,81)	1,53 (1,27–1,83)	0,68 (0,56–0,83)*	0,98 (0,81–1,17)	1,34 (1,11–1,62)

* Indicam associações estatisticamente significativas (p<0,05).

OR: odds ratio; IC: intervalo de confiança; SM: salário-mínimo, ref: referência.

Fonte: Brasil, PNS 2013 e 2019.

Em 2019, também ocorreram associações entre variáveis socioeconômicas e demográficas e às classes latentes. A classe 1 foi a de referência (Fumo, Álcool, Inatividade e Alimentação Inadequada). Na classe 2, pretos (OR 0,80; IC95% 0,62–1,01), pardos (OR 0,89; 0,75–1,05) e outras raças (OR 0,59; IC95% 0,35–0,98) continuaram apresentando menor chance, enquanto mulheres e pessoas idosas ≥75 anos apresentaram maior chance. Na classe 3 (Inatividade e Alimentação Saudável), indivíduos pretos, pardos e de outras raças apresentaram menor chance, mas as mulheres (OR 5,64; IC95% 4,61–6,90) e pessoas idosas, principalmente aqueles ≥70 anos, apresentaram chance maior. A classe 4 (Inatividade e Alimentação Saudável sem Consumo de Peixe) apresentou menor chance de pertencimento para pretos, pardos e outras raças, mas as mulheres (OR 8,08; IC95% 6,62–9,86) e as pessoas mais idosas, particularmente ≥75 anos, mostraram maior chance de pertencer a esta classe. Por fim, na classe 5 (Inatividade e Alimentação Inadequada), mulheres apresentaram maior chance (OR 6,43; IC95% 5,25–7,88), e também idosos de ≥75 anos, com menor chance de pertencimento para a raça preta.

## DISCUSSÃO

O padrão comportamental exerce importante influência no estado geral de saúde. O tabagismo, o consumo de álcool, a inatividade física e desvios alimentares refletem hábitos e estilos de vida associados a fatores de risco^17^.

No primeiro ano, destacaram-se padrões de fumo, inatividade e alimentação variada; no segundo, maior consumo de álcool e menor consumo de peixe em algumas classes. Achados de outros autores corroboram a alta prevalência de fatores de risco modificáveis no Brasil^18^.

Observou-se estabilidade na distribuição de idade e gênero, enquanto raça, escolaridade e renda apresentaram mudanças ao longo do período. As mulheres apresentaram maior probabilidade de pertencer às classes de inatividade física, com alimentação inadequada ou saudável, alinhando-se com a literatura que aponta mulheres idosas com maior inatividade, devido às responsabilidades pelos cuidados familiares e limitações físicas^19^. Por outro lado, estiveram mais presentes nas classes com alimentação saudável e tiveram menor chance de pertencer à classe com consumo de álcool, conforme evidências da literatura^19^.

O estado civil influenciou a adesão a hábitos alimentares saudáveis: idosos sem cônjuge apresentaram menor chance de pertencer às classes com alimentação saudável. Esses achados podem decorrer da ausência de apoio social e familiar^20^, que pode comprometer o bem-estar e dificultar a adoção de comportamentos saudáveis (dieta equilibrada e prática regular de atividade física). Outro achado foi a associação seletiva da maior escolaridade a comportamentos de saúde. Indivíduos com maior escolaridade tiveram maior chance de pertencer a classes com consumo de álcool — achado consistente com a literatura —, mas também maior probabilidade de integrar as classes com alimentação saudável. Apesar dessas divergências, a maior escolaridade favorece o acesso a cuidados de saúde e condições de vida mais saudáveis^21,22^.

Verificaram-se desigualdades raciais nos comportamentos de saúde que destacam desvantagens socioeconômicas e de acesso a alimentos saudáveis^23–25^. Em 2013, pretos apresentaram maior chance de pertencer à classe "Álcool, Inatividade e Alimentação Saudável sem Consumo de Vegetais e Legumes". Em 2019, pretos e pardos apresentaram menor probabilidade de integrar as classes com alimentação saudável.

Identificou-se aumento nos quintis de renda mais baixos, indicando persistência de desigualdades socioeconômicas, que podem impactar na prevalência de DCNT e seus fatores de risco^24^. Nesse contexto, pessoas idosas também apresentaram maior probabilidade de integrar classes de inatividade física, possivelmente relacionada ao declínio funcional. Evidências indicam que a atividade física contribui para maior expectativa de vida e redução das DCNT e incapacidades^24^.

Verificaram-se direções opostas na prevalência dos comportamentos aos fatores de risco avaliados. De um lado, aumentou a adesão a comportamentos positivos de saúde (consumo de frutas, verduras ou legumes e atividade física), o que pode refletir políticas públicas de saúde de promoção da alimentação saudável e prática de atividade física^24,25^. Por outro lado, observou-se a piora na prevalência de comportamentos negativos (consumo abusivo de álcool, queda no consumo de peixes) e estabilidade do tabagismo e do consumo de feijão. Essas mudanças concordam com estudos anteriores que descreveram melhora das estimativas de comportamentos de saúde positivos e piora dos negativos na população geral brasileira ao longo dos anos^18,23^.

A regressão multinomial identificou associações significativas entre fatores socioeconômicos e demográficos e as classes latentes. Nos dois anos, pretos e pardos apresentaram menor chance de pertencer à classe "Inatividade e Alimentação Inadequada com Consumo de Feijão", corroborando evidências sobre as desigualdades de acesso a recursos de saúde e alimentação^26,27^.

Nas duas amostras, confirmou-se a ausência de atividade física em todas as classes. A OMS recomenda que idosos com idade ≥60 anos façam 150 minutos de atividade física moderada por semana ou 75 minutos de atividade intensa, para manter uma boa saúde e prevenir as DCNT^27^.

Uma classe latente agrupou as características fumo, inatividade e alimentação não saudável. O tabagismo é um dos principais fatores de risco modificáveis e afeta negativamente a saúde nos diversos ciclos de vida^28^. Fumar está associado a outros hábitos prejudiciais (dieta desequilibrada, sedentarismo e consumo de álcool) que aumentam o risco de DCNT^29^. Essa junção predispõe a importantes alterações na saúde física e emocional, que causam sofrimento pessoal, familiar e alto custo social, sendo importante foco de intervenções em saúde pública^28^.

Em ambos os anos foram identificadas classes caracterizadas por alimentação saudável, associadas à inatividade física e à ausência de tabagismo e consumo de álcool. Segundo a OMS, práticas alimentares saudáveis iniciadas precocemente geram benefícios duradouros à saúde dos indivíduos^30^.

O consumo alimentar saudável é determinado por posições socioeconômicas e demográficas. O Brasil está entre os países mais desiguais e com elevados níveis de má nutrição na população em geral^31^. Estudo com dados da PNS indicam maior consumo de alimentos saudáveis entre indivíduos com maior renda e escolaridade, grupo que também apresentou maior prevalência de consumo abusivo de álcool, evidenciando a coexistência de comportamentos protetores e de risco^5^.

Assim, o agrupamento dos fatores de risco modificáveis à saúde reflete complexa junção de fatores biopsicossociais e raciais. A simultaneidade dos fatores sugere que intervenções para a promoção da saúde devem ser multidimensionais, não abordando fatores de risco de forma isolada. Logo, estratégias para comportamentos de saúde sinérgicos devem considerar os determinantes sociais que são fundamentais na adoção e manutenção de comportamentos saudáveis.

Os achados reforçam a importância da adoção e manutenção de comportamentos saudáveis, indicando que os padrões de risco resultam da combinação de hábitos de vida. Esses padrões influenciam o cuidado com a saúde, o engajamento social, o acesso a ações e serviços, e a interação entre indivíduo e ambiente. Evidências internacionais corroboram essa perspectiva. Estudo realizado na Tailândia identificou padrões alimentares inadequados e sobrepeso/obesidade como os principais fatores de risco para DCNT, destacando a necessidade de ações voltadas à redução das iniquidades^32^. Na África, apontou-se que DCNT e a saúde mental estão associadas à interação de fatores ambientais, comportamentais, biológicos e sociais^33^. As semelhanças com o contexto brasileiro incluem a persistência de iniquidades e barreiras estruturais que limitam o acesso aos serviços de saúde, medicamentos e às tecnologias para o manejo das DCNT.

No Brasil, políticas públicas como a Política Nacional de Saúde da Pessoa Idosa, o Guia de Cuidados à Pessoa Idosa, o Estatuto da Pessoa Idosa e o Plano de Ações Estratégicas para o Enfrentamento das DCNT fornecem bases importantes para a promoção do envelhecimento saudável e a redução das desigualdades. A Política Nacional de Vigilância em Saúde reforça a centralidade do território e da equidade no cuidado às populações vulneráveis, elementos diretamente relacionados aos achados deste estudo^34^.

Por fim, destacam-se as limitações metodológicas. Os comportamentos analisados não abrangem todo o espectro do estilo de vida; os dados são autorreferidos, sujeitos a vieses; e a LCA é exploratória, dependente da seleção das variáveis e não permite inferências causais. Mudanças metodológicas entre as edições da PNS também podem ter influenciado as prevalências observadas.

Apesar dessas limitações, os resultados contribuem para o planejamento de ações de cuidado, vigilância e promoção da saúde, oferecendo subsídios para intervenções voltadas à redução de comportamentos de risco combinados e à promoção do envelhecimento ativo e saudável.
